# Bridging academic science and clinical research in the search for novel targeted anti-cancer agents

**DOI:** 10.7497/j.issn.2095-3941.2015.0079

**Published:** 2015-12

**Authors:** Alex Matter

**Affiliations:** Experimental Therapeutics Center & D3, A*STAR, Singapore 138669, Singapore

**Keywords:** Drug discovery, drug development, translational R&D, targeted anti-cancer agents

## Abstract

This review starts with a brief history of drug discovery & development, and the place of Asia in this worldwide effort discussed. The conditions and constraints of a successful translational R&D involving academic basic research and clinical research are discussed and the Singapore model for pursuit of open R&D described. The importance of well-characterized, validated drug targets for the search for novel targeted anti-cancer agents is emphasized, as well as a structured, high quality translational R&D. Furthermore, the characteristics of an attractive preclinical development drug candidate are discussed laying the foundation of a successful preclinical development. The most frequent sources of failures are described and risk management at every stage is highly recommended. Organizational factors are also considered to play an important role. The factors to consider before starting a new drug discovery & development project are described, and an example is given of a successful clinical project that has had its roots in local universities and was carried through preclinical development into phase I clinical trials.

## Introduction

Drug discovery and drug development have undergone major and, one is tempted to say, revolutionary changes. The siloes of industrial and academic research have been opened, largely as a result of stagnation in the pharmaceutical industry too long focused on minor improvements of block-buster medicines and huge marketing armies of sales representatives^1^. The industry had to reinvent itself, and those companies who were able to do so survived^2^^,^^3^. Many companies disappeared—swallowed by more powerful rivals, with devastating effects on overall productivity^4^. At the same time companies had to look out for new sources of innovation to rejuvenate their aging pipelines. Academia as the pioneer, and small biotech became the major drivers in this effort^5^. At present, a young and vigorous biotech industry is dominating the field of innovative novel medicines, medicinal technologies and analytics^6^. Genomics has made its powerful entry, and clinical trial methodology has been revamped based on entirely new algorithms, often summarized under the term “Precision Medicine”^7^^-^^10^. Cancer medicine is in the forefront of these developments, with some very negative consequences, such as the overpricing of the most modern novel anti-cancer drugs^11^. This has led to a backlash of the public, the payers and health authorities. It will take several years, and possibly a decade to find a balance between patient benefit afforded by novel medicines and a fair pricing model.

## 1990-2010—emergence of targeted drugs—the triumph of reductionism

The nineties saw the emergence of targeted anti-cancer drugs such as trastuzumab (HerceptinR)^11^^,^^12^, the first monoclonal antibody, and imatinib (Glivec/GleevecR)^13^^,^^14^, the first kinase inhibitor. These were two pioneering breakthroughs. It is often forgotten that before these discoveries all-trans retinoic acid (ATRA) was found to be active against 5% of acute promyelocytic leukemia (APL), a finding made in Shanghai by Chinese investigators, Wang and Chen, already in 1985^15^. ATRA works in APL because of a chromosomal translocation of chromosomes 15 and 17 causing a genetic fusion of the retinoic acid receptor (*RAR*) gene to the promyelocytic leukemia (*PML*) gene. While this precise mechanism of action was unraveled only several years later, in the case of trastuzumab and imatinib it was primarily the new conceptual approach that pushed the drug discovery effort.

Indeed, targeted drugs address one or more well-defined biochemical targets (enzymes, receptors, transcription factors), usually in the context of deregulated signal transduction pathways^16^^-^^18^. Targeted drugs address homogeneous patient populations with regard to the target and allow for molecular diagnostics for patient selection/follow up. Targeted drugs must yield pharmacodynamic readouts in early clinical trials (biomarkers)^19^^,^^20^ and are expected to offer enhanced safety because of their selectivity.

Not all of these expectations were met by reality. Targeted drugs can show toxicity, and often do not show ideal selectivity. This can play into our hands since a several targets can be hit by one drug such as imatinib^13^^,^^14^. Imatinib can inhibit e.g., gastrointestinal stromal tumors (GIST)^21^^,^^22^. This inhibition is achieved through c-kit receptor inhibition that is constitutively activated in this tumor^23^^,^^24^. There are many more cases like this. Another famous example is crizotinib which was designed as a c-met inhibitor but which was most successful in inhibiting ALK+ NSCLC^25^^,^^26^. By now, there are more than 25 targeted drugs approved for treatment of various indications^27^. As a general feature it can be stated that most targeted drugs work best on mutated and constitutively activated targets.

Targeted drugs have changed cancer medicine and have given thousands of patients increased progression-free survival (PFS)^28^. Improvement of overall survival (OS)^28^ remains an elusive goal, though, with the notable exception of Glivec^29^. In recent years combinations of targeted drugs were tested in the clinic, often with quite surprising results regarding toxicity^30^. The toxicity pattern of combinations of targeted drugs is quite different from that observed with cytotoxic drugs. Sometimes severe toxicity is found in the vascular system by treating patients with ponatinib, a Bcr-Abl inhibitor being active against the notorious T315I mutation of the Abl kinase^31^.

## 2010-2015—return to more holistic approaches—emergence of immunotherapy, cellular therapies and other novel modalities

The attempts to find drugs stimulating the immune system to eradicate cancers go back several decades, mostly with disappointing results. Also, the search for drugs alleviating an immune suppression triggered by the tumor itself has a long history. But it is only with the much more profound understanding of immune mechanisms that at last success was at^32^. Indeed, a successful immune therapy was achieved with monoclonal antibodies blocking some of the brakes that the immune system triggers preventing an effective anti-tumor response to eliminate tumors such as advanced melanoma^33^^,^^34^ and NSCLC^35^. Two modalities are standing out with their clinical successes, admittedly in a minority of patients: anti-CTLA4 (ipilimumab) and anti-PD-1/PD-L1 monoclonal antibodies such as nivolumab and pembrolizumab^36^. Additionally, a cellular modality, a chimeric antigen receptor T cell (CAR-T)^37^ based on transformation of autologous T cells, has garnered a lot of attention after the recent reports of some impressive clinical trial results^38^.

## The place of Asia in modern drug discovery and development

Close to 60% of the world population (7.2 billion in 2015) live in Asia with a huge diversity in ethnicities, religions, history and income/capita. The huge continent is fragmented through geography and a near infinite variety of political systems. Six out of ten countries are on the list of the world’s most populous countries with China and India topping the list^39^.

The history of medicine in Asia is strongly imprinted by its emphasis on traditional medicines that maintain their role as a primary source of medicines in most Asian countries^40^^,^^41^. This may have influenced the growth of Western-based medicines and pharmaceutical industries in the region. Big multinational companies as well as start up’s in the Western tradition are rather the exception or are lacking entirely. The top 20 pharmaceutical companies by revenue (2014) only comprise two Japanese companies (Takeda #10; Otsuka #18)^42^. No Chinese or Indian companies are found among these top companies. This is certainly not due to a lack of scientific talent, capital and other resources. It has more to do with infrastructure, talent pools, specialized expertise and a lack of translational research linking state-of-the-art basic research into disease mechanisms with sophisticated clinical research.

## The complexity of biomedical R&D

R&D into novel medicines is indeed a major challenge since it depends on macroeconomic and societal factors as well as a convergence of local enablers that can trigger the emergence of a flourishing biotech industry. The entirety of these factors is often understood as an “ecosystem”. At the outset, it should be noted that the emergence of a profitable and sustainable biotech industry is not a linear process and that non-linear, often chaotic events can play a very favorable or deleterious role. Critical mass plays a critical role and in practically all cases of successful developments we see geographically confined sites—cities and relatively small regions—as the birthplace of innovation. Innovation in this context means products and services that effectively cover the needs of customers who are willing to pay, or the insurance of whom is willing to defray the costs. Needs arise in healthy people (for diagnostics, diagnostic procedures, vaccines, preventative medicines, diets or food) and in patients (again for diagnostics, therapeutic vaccines and medicines, and interventions relying on medtech instruments).

Innovation is almost always preceded by a discovery stage, leading to translational research and development, followed by registration (in most biomedical applications) and marketing of the final product and/or service. This sequence of discovery and development requires many diverse skill sets that need to contribute at the right time, in an optimal way to a successful product and it is the management and orchestration of these processes that drug discovery and development falters. Additionally, the whole process extends over at least 10 years in the case of novel medicines, is very expensive and fraught with failures^43^^,^^44^. The failure rate, i.e., attrition is something that distinguishes R&D of the pharmaceutical industry from all other industries. It would be unthinkable to accept a 90% failure rate in engineering projects such a bridge building, for example. This factor alone poses a very high entry barrier that discourages many governments, universities and investors and possibly represents the single most important selection factor for any fledgling effort to start a new venture. On the other hand, risk aversion, which is inherent in all larger organizations, is a protective baseline of human behavior.

High risks must be offset by high rewards. These rewards are in many cases financial (as reflected by the spiraling cancer drug prices) or rewards offering protection against a clear and present danger. Extreme cases for the latter was HIV/AIDS, perceived to be threatening humankind in its entirety at its emergence, or much more recently, the Ebola fever threatening to get out of control.

There is a middle ground where vaccines and medicines have effectively contributed to longer life expectancy through the advent of antibiotics, anti-diabetics and statins as poignant examples. Alternatively, medicines have contributed to a major improvement of the quality of life with antidepressants, analgesics and antipsychotics as prime examples.

In other fields of medicine the scorecard is mixed, or even unsatisfactory, and cancer is unfortunately one field of medicine where progress is earned the very hard way, going forward step by little step. We have made enormous progress, certainly, but it took decades to achieve and we are not where we want to be. Currently, about two thirds of all cancers are curable (expressed as 5-year relapse-free survival) which represents major progress^45^; however, it is also often said that 75% of all cancer patients do not benefit from treatment.

The major and biggest obstacles to better results can be grouped into preventable cancers through diet and vaccinations, and cancers that would be curable if they were detected earlier. Preventable cancers include:

- Smoking: reduction of smoking would be the single most effective measure to reduce incidence and mortality, primarily of lung cancer^46^;
- A variety of other dietary factors including e.g., aflatoxin contamination of peanuts^47^, parasite infection (*Opisthorchis viverrini*) by fish in North-East Thailand, causing cholangiocarcinoma^48^, or betel nut chewing in cancers of the oral cavity^49^, and others;
- Lack of vaccination against cancer-causing agents, e.g., anti-HPV vaccines for the prevention of cervical cancer^50^ and other genito-urinary tract cancers, or anti-HBV vaccination for the prevention of liver cirrhosis and liver cancer^51^.

Cancers that would be curable if they were diagnosed at an early stage include:

- Major solid cancers including hepatocellular carcinoma, pancreatic and gastric carcinoma, lung cancer and to some extent, colon and brain cancer.

Especially in developing countries, prevention and early detection would certainly be among the most cost-effective solutions to the pressing need to deal with what has been called a “cancer epidemics”^52^. Unfortunately, a very small percentage of resources, comparatively speaking, are devoted to these two major challenges.

Cancer R&D is a field where the very high financial rewards attract a lot of investment, but where the needs of Public Health are often relegated to a distant second place. This is increasingly leading to tensions between the two sectors, with pricing usually being the focal point of discord. Different countries and regions are attempting to find a balance between the needs of investors turning a profit from their high-risk, long-term investments and the needs of the Public Health sector to protect its people from early disease and death by the use of cost-effective, safe and efficacious medicines^53^^,^^54^.

## New models of open-sourced R&D

In countries without a well-established pharmaceutical industry one needs to look at new models of R&D. To establish a major pharmaceutical industry takes several decades at least but the medical needs of many Asian countries are pressing and in many cases specific to a particular country or region, and Western medicines are in many cases either too expensive or not suited to cover specific medical needs. As an example we can cite the recently approved anti-viral medicines for treatment of HCV that are very effective against hepatitis C^55^ (and then contribute to prevention of hepatocellular cancer induced by this virus), but staggeringly expensive^56^. Another example is Dengue fever, with a huge annual incidence of up to 500,000 cases, where an effective vaccine is still missing despite huge efforts of industry and academia^57^.

New models of R&D need to be developed to create novel medicines in countries and regions without a long tradition of drug discovery and drug development expertise. There are several options:

- Established pharmaceutical companies are invited to set up subsidiaries in the less-developed or developing, and are incentivised to work on diseases that are relevant to that particular country or region. Examples for this can be found in China, Singapore and other countries;
- Established multinational pharmaceutical companies (MNC) often scout academic centers for novel drug targets, diagnostic/medtech application and other technologies. This is often followed by investment of the MNC in local research and development groups;
- Investors are invited to set up shop close to academic centers to build and establish start-up companies, often jointly funded by government and investment groups, with either local or international intellectual property;
- Governments develop R&D strategies that aim at fostering a local ecosystem that enables start-up companies to be formed. Generally, this takes place in “incubators” where young scientists with entrepreneurial bend can try out novel concepts with small funding streams, grants or loans;
- Governments go further in establishing large basic research and translational R&D facilities with the specific aim to generate economic benefit through generation of intellectual property that can be licensed out, establishing spin-off’s, and high-value jobs creation. Riken in Japan, and A*STAR in Singapore are two Asian examples of this approach.

All of these options rely on the presence of large, highly ranked universities with their talent pool and expertise in a multiplicity of disciplines. On their own, however, universities will rarely generate truly successful young companies. In most cases, these ventures will remain small and tentative, and not achieve a place in the global market. This must be said to be eminently true in almost all Asian countries. In summary, it is fair to state that the record of above initiatives is mixed.

The question then arises what is needed beyond infrastructure, talent pools of universities, financial resources and intellectual property to do what cities like Boston, San Diego, San Francisco, Oxford/Cambridge/London and Munich have achieved? Why is the goal of translating basic research into approved and marketable products and services so elusive, in particular in the biomedical sector?

## Success factors for the creation of a new biomedical industry

Clearly, we have environmental factors like infrastructure, transport, telecommunication, security, language hurdles, openness to foreign talent and willingness of the society, i.e., the government as well as the private sector to invest heavily and over the long-term. A talent pool of well-educated scientists and engineers is required with people who are willing to forego the security of, say, a government-funded research laboratory stepping into the risky and harsh environment of start-up’s. It is often forgotten that translational research is a much disciplined activity, comprising often large, multi-disciplinary teams (especially at an advanced stage) the composition of which must constantly adapted to the changing needs of a project. One of the major hurdles is to combine the individually specific expertise of team members in a complementary team that can function effectively, and sometimes over years. Moreover, it is often painful to see a project fail, when decisions need to be made to terminate a project, often based on an incomplete data set, and despite the hope of the project champions, that the project might still see the light of the day.

To be effective, we need to establish a division of labor as follows:

- Basic research driving target discovery, target validation, prototype assays, target epidemiology and biomarker discovery;
- Discovery, comprising (for small-molecular weight approaches) assay development, screening, hit-to lead finding and lead optimization, pharmacokinetics, efficacy studies and preliminary toxicity studies. For large molecules (antibodies and related biologics) we would recognize epitope definition, prototype antibodies, optimization and humanization (as the case may be), antibody engineering, receptor engineering as key activities;
- Preclinical development usually comprises upscaling according to good manufacturing practices (GMP)^58^, stability and purity testing, process chemistry (for small molecular weight compounds), salt screens and formulation, regulatory toxicity studies according to good laboratory practices (GLP)^58^, usually in at least two animal species, and pharmacokinetic studies looking at absorption, distribution, metabolism and excretion (ADME), also according to GLP^58^.
- Early clinical trials in man: for anticancer compounds healthy volunteers can only be used in exceptional cases. Even the very first studies, looking at tolerability and dose-limiting toxicity are usually done in patients. In the field of cancer, the clinical trial paradigms have shifted in a major way: in many cases, the very first studies are followed by an expansion phase, using Bayesian, adaptive designs, and prognostic biomarkers for enrichment of patient populations with patients who would be predicted to respond, and eliminating, if possible, those patients who would be predicted to be unresponsive. Predictive biomarkers are used to demonstrate target engagement within the patient, either in the tumor itself or in surrogate tissues (blood cells, hair follicles, buccal swabs).

The overall aim of the early clinical trials is to attain a Proof-of-Concept (PoC) in man, which comprises the following elements:

- Availability of a stable formulation of a pure compound or biologic entity;
- Satisfactory tolerability, when balancing risk-benefit for the patient;
- Adequate pharmacokinetics in terms of C_max_, half-life, tissue distribution and overall bioavailability;
- Positive read-out for biomarker activity, i.e., target engagement;
- Early clinical read-out’s such as tumor shrinkage (not relevant, e.g., for immunotherapeutic approaches where an increase of tumor size can be observed before ultimate shrink and disappearance).

## Organizational challenges

From the above, the complexity of translational biomedical R&D becomes quite apparent and a division of labor is the only way to deal with it. At our own facilities in Singapore, we have divided the tasks as follows.

**Figure 1:**
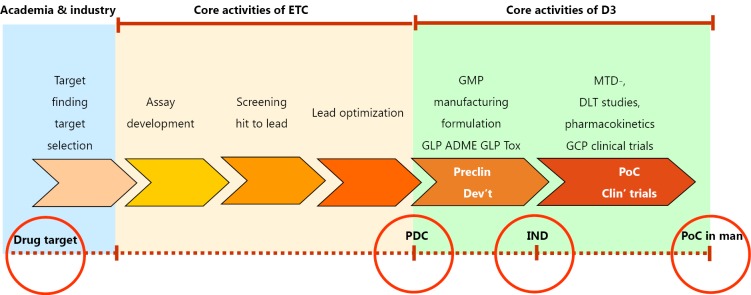
The flow of activities from a validated drug target to discovery (done at ETC) and early development (done at D3). Each phase is comprising several stages. Discovery of small molecular weight compounds usually proceeds from assay development and screening to hit-to-lead and then to the lead optimization phase. In early development we distinguish preclinical development with up scaling and GMP quality manufacturing, formulation, stability, GLP tox and ADME profiling; in early clinical trials we usually have a phase Ia for tolerability and pharmacokinetics; in phase Ib we often see an expansion phase where patient populations are enriched with patients who are likely to respond based on genomics data. GCP, Good Clinical Practice; MTD, Maximally tolerated dose; GMP, Good Manufacturing Practice; PDC, Preclinical Development Candidate; GLP, Good Laboratory Practice; PoC, Proof-of-Concept; DLT, Dose-limiting toxicity; ADME, Absorption, Distribution, Metabolism, Excretion.

**Figure 1** explains the flow of activities at our facilities in Singapore, the Experimental Therapeutics Center (ETC) for the upstream activities, and Drug Discovery & Development (D3) for the downstream preclinical development and early clinical trials:

- Basic research (target discovery, target validation, #1) is expected to come from the academic sector, or in some exceptional cases, from industry;
- Discovery (#2) is done at the ETC of A*STAR, exclusively for small molecular weight compounds;
- Preclinical Development (#3) and Early clinical trials (#4) are performed by D3 (Drug Discovery & Development) of A*STAR;
- Biomarker discovery is done through a separate grant mechanism (“Companion Dx in Cancer”, CDIC).

While the division of labor is unavoidable, it is also evident that this creates multiple interfaces and resulting communication hurdles. These communication flows are all the more important as the different segments of biomedical R&D speak different “languages” with their own jargon, technical terms and a host of acronyms that are impregnable to the outsider. It is then crucial to have senior managers who can speak these languages building bridges between the various communities. It takes the team members usually several years and patient training to learn how to effectively communicate within the team, and across teams.

To address this complex translational R&D we need to work with organizational structures that are far removed from groups centered on a Principal Scientist. In our experience, translational R&D is best done in a matrix organization, with skill groups and project teams drawing resources from the various skill groups in line with variable project needs.

**Figure 2:**
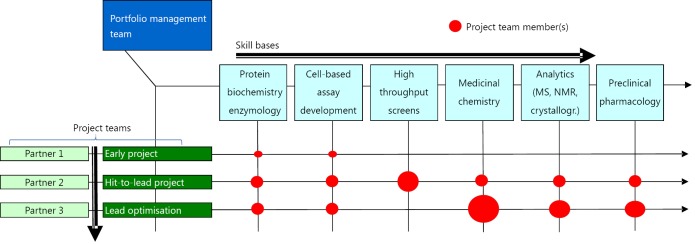
Matrix organization.

**Figure 2** illustrates the lifecycle of a project in discovery pointing out that in quantitative as well as qualitative terms a project is subject to constant changes. This makes the management of these multidisciplinary projects much more demanding than, say, the management of a theme-centered basic research group.

## Specific points to consider before starting a new R&D project

### Scientific rationale

At our facility in Singapore, we ask minimally the following questions regarding the scientific rational of a new project:

Causality chain between a deregulated/mutated drug target and disease phenotype explained through an understanding of molecular pathophysiology;

- Epidemiology of the disease pathophysiology—frequency of deregulated/mutated drug target in a particular disease;
- Drug ability of the drug target via small molecular weight compounds, antibodies, antisense, vaccines, cellular therapy, gene therapy. Is a crystal structure available? Would a structural analysis by protein NMR approach be feasible? Is a fragment-based screen feasible/desirable?
- Primary assays: description of the assay format— biochemical screen or phenotypic screen in reporter cell assays. Can it be reproduced in our laboratories? Is it likely that it can be formatted for a high-throughput screen.

**Figure 3:**
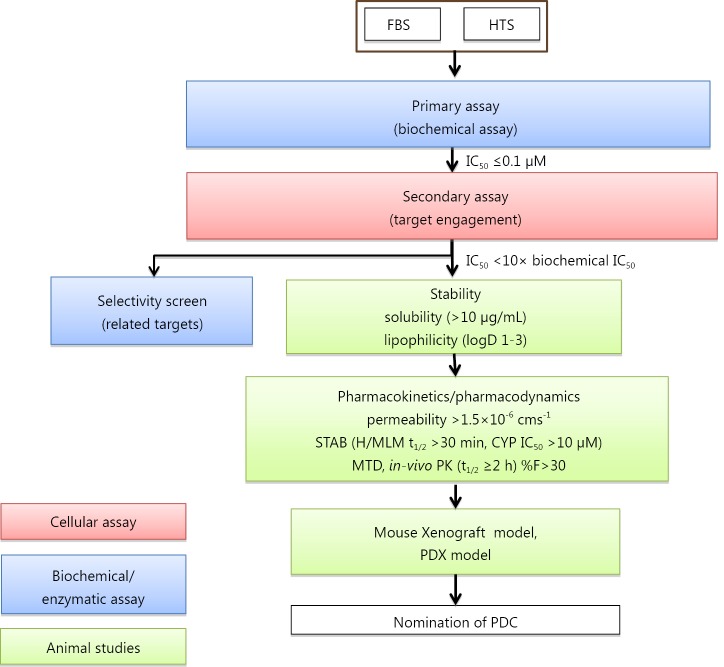
The screening cascade of a typical project pursuing a small molecular weight compound-based approach. FBS, fragment-based screen; HTS, high-throughput screening; PK/PD, pharmacokinetics/pharmacodynamics; PDC, preclinical development candidate.

Usually, we allocate a 6-month period for exploratory activities, primarily to check the robustness of the assays, the literature, patent status and competitive position and building a robust flow chart (**Figure 3**).

### Medical need

- Precise description of a disease type, with a specific pathophysiology;
- Example: “Breast cancer” is not a disease, but a collection of many diseases;
- “Trastuzumab-resistant HER2+ advanced breast cancer” may be a homogeneous disease, but may still contain several subtypes.

In recent years, the genomics definition of a cancer type has acquired an accepted position. This means, however, that tumor types such as breast cancer are now split into ten clearly recognizable subtypes^59^ and gastric cancer^60^ into at least six genetically distinct subtypes. Inevitably, this will lead to a further splitting of major diseases into more and more subtypes, with the foreseeable arrival of patient-centric (N=1 clinical trials) profiles. It will remain one of the major challenges of future cancer medicine to deal with the

- Heterogeneity of cancer disease and its many subtypes;
- Heterogeneity within a primary tumor;
- Constant emergence of new clones and disappearance of old clonal subtypes in one cancer patient.

Immune oncology may offer a path out of this bewildering diversity addressing—when it is successful—a tumor in its entirety and producing complete responses.

### Experimental approach—flow chart/screening cascade

The screening approach should be carefully considered upfront; small pilot screens are often used to test for the feasibility of a given approach. The characteristics comprise:

- In general going from simple, high-throughput systems to complex, low throughput systems);
- High throughput systems are fast, relatively cheap (per assay point) but have little predictive value;
- Low throughput systems are generally labor-intensive, expensive and slow but should have high predictive value.

The screening approach should ideally allow identifying a preclinical candidate with a minimum of resources, within a competitive time frame. A Flow chart is then built as follows.

### Competitive position

Finally, the competitive position is important to consider. Do you have a competitive niche? Can you differentiate yourself from competition? The questions are:

- Who are your real or suspected competitors?
- Understand direct and indirect competition;
- Direct competition: same target, different compound;
- Indirect competition: different pathway, or different approach altogether (e.g., surgery instead of drug treatment);
- Are you in a lead, mid-field or rear position?
- Document your statements by literature, with the help of consultants, and through the “grape-vine”.

### Regulatory aspects

Regulatory aspects need to be understood at the outset and where this expertise does not exist, it needs to be brought in from the outside. Failure to comply with regulatory guidelines can be fatal. A first major decision point is the submission of the dossier to request permission to enter clinical trials. Key documents include the Chemistry-Manufacturing-Control (CMC) dossier, the investigator brochure (IB), the Informed Consent Form and the planned Trial Protocol. The overriding consideration is safety, in all cases. In most cases it is recommended to entertain a detailed dialogue with the Health Authorities to get a good understanding of key issues. Equally important is the permission from the Ethical Review Board to expose patients of healthy volunteers to the drug. Local conditions vary but again, safety issues have highest priority.

### Marketing

Market analysis is a valuable tool if used with caution and by people with a lot of experience. Market analysis starts with the analysis of epidemiological parameters such as incidence and prevalence, and how well a medical need is covered by standard-of-care (SoC) treatment. The new modality needs then to be compared with SoC and likely patient benefit estimated in terms of OS and quality of life. The competitive position of the new modality then needs to be positioned with direct and indirect competitors, at the time of market entry (!) and market penetration estimated. Pricing and reimbursement issues will heavily influence penetration.

It is evident that most figures are based on estimates and can be wrong. Market estimates are more precise in the case of improved versions of existing drugs; for truly innovative medicines most marketing estimates are far from the eventual figures achieved in the market. Lastly, it must be remembered that price setting in many countries is tightly regulated by health ministries or payers from the insurance industry. In the end, some form of compromise must be found between manufacturer and payer.

## An example of a successful anti-cancer project—Wnt/porcupine inhibitor

This example illustrates the successful R&D in the area of the Wnt pathway landscape, itself a most complex area that was thought non-druggable for a very long time. Increased, abnormal Wnt signaling promotes tumorigenesis by driving cellular proliferation, blocking differentiation, promoting epithelial mesenchymal transitions, and promoting both stem cell renewal and metastasis. There are 19 Wnt ligands and 10 Frizzled receptors signaling through at least three major pathways. To cope with this complexity and to discover useful drug candidates has remained a riddle until the discovery of a single vulnerability.

All mammalian Wnt ligands require posttranslational modification. A single essential, nonredundant enzyme, PORCN (the human ortholog of the Drosophila gene porcupine) adds a mono-unsaturated palmitoleate moiety to a serine residue conserved in all mammalian Wnts. This palmitoylation is essential for both Wnt secretion, and the binding of Wnts to their receptors. Thus, inhibition of PORCN blocks the secretion and activity of all human Wnts. This opened the door to the discovery of selective and potent inhibitors of PORCN^61^.

**Figure 4:**
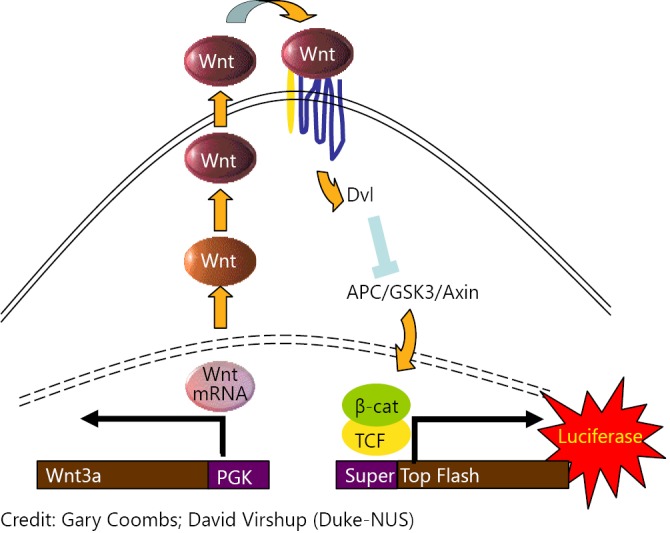
STF3A cells.

Since there was no direct biochemical assay available at the time of starting the project, we relied on a cellular (phenotypic) screen to identify hits that could serve as a starting point for a hit-to-lead finding, and ultimately, to lead optimization (**Figure 4**). To identify potent inhibitors of Wnt secretion, we screened a library of ~225,755 small molecules using a multi-step cell-based screen. HEK293 cells with constitutive high Wnt/β-catenin signaling due to stable expression of WNT3A and harboring a luciferase-based Wnt/β-catenin reporter (Super 8xTOPFLASH) (STF3A cells) were incubated with small molecules for 24 h. Luciferase reporter activity was used as a measure of Wnt-pathway activity. To specifically identify inhibitors of Wnt secretion, potent compounds that were not cytotoxic were tested using a HEK293 cell line with an integrated STF reporter plasmid (STF cells) and exogenously supplied WNT3A conditioned medium.

**Figure 5:**
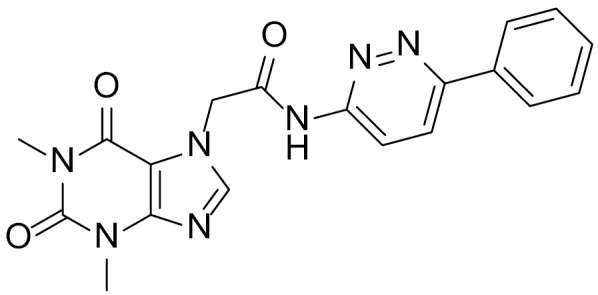
ETC-159 (phase I clinical trial, June 2015).

Compounds that selectively inhibited signaling in STF3A but not in STF cells with WNT3A conditioned medium and had an IC50 of <1 μM were selected as PORCN hits and progressively optimized to yield the compound ETC-159 (**Figure 5**).

Upstream PORCN inhibitors are not expected to be of benefit in Wnt-deregulated cancers with downstream mutations such as APC, AXIN, GSK3, or β-catenin mutations. However, there are a number of upstream mutations where the Frizzled receptors are deregulated that can be shown to be susceptible to PORCN inhibition. These include RNF43^62^, ZNRF3 loss of function^63^^,^^64^ and R-spondin (RSPO) gain of function through translocations^65^^,^^66^ and Notch 1, 2 and 3 loss of function^67^. These may be excellent predictors of PORCN efficacy. While these mutations are not very frequent, they occur in a wide variety of solid cancers primarily of the gastro intestinal and genitourinary tract.

The clinical trial strategy has to adapt to this epidemiology. The recently developed strategy of “Basket trials”^68^, i.e., testing one drug target in a genetically defined fraction of many different cancers is a methodology that can hopefully cope with this difficulty. Prognostic biomarkers in the expansion phase of this trial will be RNF43/ZNRF3 and R-SPO translocation for patient enrichment, while patients with APC, AXIN, GSK3 and β-catenin mutations will be rather dissuaded from joining this particular trial. Predictive biomarkers will determine target engagement, likely in peripheral blood mononuclear cells (PBMC) or better in hair follicles, and the biomarker activity in patients will be correlated with early clinical readout’s, i.e., tumor shrinkage. A waterfall plot will then be constructed to illustrate the degree of correlation. A significant response rate according to RECIST criteria^69^, correlating with biomarker activity will constitute a PoC and lay the basis for regulatory, pivotal clinical trials. There is currently a vigorous debate going on whether an extended trial showing good correlation between response rate and biomarker activity is in itself the basis for at least a conditional approval or whether traditional phase III trial designs should still be used for regulatory submission. In the cancer arena, the ground is shifting, especially when patient benefit is high and indications are small.

For interest, it may be noted that an alternative new clinical trial paradigm has been developed, the so-called “umbrella trial”^70^. In this situation, contrary to the Basket trial, the study cohort consists of one well-defined clinical indication. The members of the study cohort will be subdivided into groups and allocated to different treatment regimens that are most suitable for their particular cancer. Patients with cancers that cannot be allocated to new treatment regimens based on “actionable” targets (i.e., targets with compounds on the market or in advanced clinical trials) will receive Standard of Care (SoC) and serve as controls. Groups that show no or negligible response rates will be closed down (“futility”); groups with promising response rates will be enhanced in an adaptive design.

## Productivity of public sector-driven, open R&D in cancer

The above gives an overview on a new way of driving R&D in areas that are of a particular concern to countries and regions, and where countries cannot rely on a well-established and competitive pharmaceutical industry.

In our facilities in Singapore, we are working with approximately 90 FTEs plus about 25 outsourced FTEs at ETC, in collaboration with academic centers such as A*STAR institutes, Duke-NUS, the National Cancer Center, and others. Some projects are done in international partnerships. We have been following this paradigm for the past 6 years and are now more or less at steady state, producing on average two preclinical development candidates (PDCs) each year. We think that this is a competitive performance provided that the PDCs are of high quality and have acceptable attrition rates until the commencement of clinical studies via an IND (CTC in Singapore). We would think that an attrition rate of 50% is acceptable.

In D3, we have about twelve experts with longstanding pharmaceutical/clinical trial experience, a range of specialized consultants and the resources to drive a portfolio of 5-6 compounds in preclinical development or early clinical trials. Most technical activities are outsourced; clinical trials are done regionally or internationally. We are striving very hard to produce one IND/CTC each year, again an ambitious goal.

Overall, we think that this level of productivity is at least on a par with highly competitive pharmaceutical companies.

## Conclusion

We have seen above that there are many serious or even severe medical needs that are either not covered due to scientific/technical hurdles, or are neglected for lack of commercial incentives. We believe that the public sector, with its universities, research facilities and hospitals can be a major player to alleviate pressing medical needs, to foster R&D in neglected areas and to build a counterweight against sometimes predatory pricing in some of the pharmaceutical industry.

The major rate-limiting factors are mostly well known: infrastructure, health services, an ecosystem with a talent pool, expertise in many different disciplines and capital from the public and/or private sector. Less well recognized are factors such as the recognition of the crucial importance of translational research and development, the disciplined approach that is necessary to drive discoveries to innovation and the willingness to accept risk and failures as unavoidable companions on this voyage.

Asia can play a more important role and be on par with the best of Western pharmaceutical companies. Japan has been a pioneer and is on its way; a similar situation prevails in Korea. Other nations, including India and China have all the ingredients but still have some way to go to be comparable with the best.
